# Successful Escape of Acute Ischemic Stroke Patients from Hospital to Home: Clinical Note

**DOI:** 10.3233/BEN-2012-0357

**Published:** 2012-03-16

**Authors:** Hideaki Tei

**Affiliations:** Department of NeurologyToda central General HospitalSaitamaJapan

**Keywords:** Acute ischemic stroke, escape from hospital to home, aphasia

## Abstract

I describe four patients who successfully escaped from the hospital to their own home during the acute phase of ischemic stroke. This is a very rare phenomenon (seen in 0.35% of 1150 consecutive patients with first ischemic stroke within 24 h after onset), but the patients had rather uniform clinical characteristics. All were male, around 60 years old, had moderate to severe aphasia (Wernicke’s in 2 patients, Broca's in 1, and transcortical motor in 1), and cerebral infarction of the left middle cerebral artery territory. None had significant motor weakness, hemispatial neglect, or hemianopia at the time of escape. Overall functional outcome was good for all but one patient, but aphasia persisted in three. Although none of the four patients sustained serious injury during the escape, patients with such clinical characteristics must be managed cautiously to prevent serious consequences.

## 1. Introduction

In acute stroke patients, mood and behavior disorders are very frequently observed. Among them, overt sadness, disinhibition, lack of adaptation, environmental withdrawal, crying, anosognosia, passivity, and aggressiveness are common change shown on the Emotional Behavioral Index [1]. Some patients with denial behavior may be unable to comprehend that they are unwell and try to get out of bed and go home [1]. Usually, this behavior is detected by medical staff, but it can result in falls or even escape from the ward or hospital [4]. I encountered four patients who successfully escaped from the hospital to their own home while hospitalized for acute ischemic stroke. Although they did not experience any harm during the escape, serious injuries can result in these circumstances.

Of 1150 consecutive patients with first acute ischemic stroke who were admitted within 24 h from onset to the Department of Neurology, Toda Central General Hospital, Saitama Prefecture, Japan, from May 1994 to August 2010, four patients (0.35%) escaped from the hospital and returned home. All incidents had happened during hospitalization in a general ward. Summary of these patients are presented in Table 1. Aphasia was assessed using the aphasia quotient of the Western Aphasia Battery and graded as severe (scores 0–39), moderate (scores 40–79), or mild (scores 80–93.7) [7]. Patients had rather uniform clinical characteristics. All were male, around 60 years old, had moderate to severe aphasia (Wernicke's in 2 patients, Broca's in 1, and transcortical motor in 1), and cerebral infarction of the left middle cerebral artery (MCA) territory, while none had significant motor weakness, hemispatial neglect, or hemianopia at the time of escape. Overall functional outcome was good (modified Rankin Scale $\leqslant 2$) for all but one patient, but aphasia persisted in three. None of the four patients sustained serious injury during the escape.

The clinical course of the most illustrative case is as follows (Table 1. number 4).

A 60-year-old man was brought to our hospital by ambulance 7 h after suddenly developing faintness and inconsistent speech during a meeting. He had no medical or psychological history. He was somnolent but would awake to loud voice or ordinal pain. There was

ISSN 0953-4180/12/$27.50 © 2012 – IOS Press and the authors. All rights reserved

Table 1
Summary of 4 patients

| Patient Number | Age/Gender | NIHSS score at onset | NIHSS score at escape | Type of aphasia | Timing of escape (after onset) | mRS score ( after 3 months) | Lesion |
|---|---|---|---|---|---|---|---|
| 1 | 66/male | 6 | 6 | Wernicke's | 4 days | 1 |  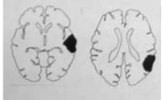 |
| 2 | 60/male | 17 | 9 | Broca's | 17 days | 3 |  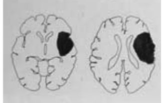 |
| 3 | 60/male | 5 | 5 | Wernicke's | 7 days | 1 |  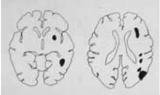 |
| 4 | 60/male | 6 | 4 | Transcortical motor | 5 days | 1 |  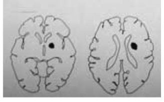 |

NIHSS: National Institutes of Health Stroke Scale mRS: modified Rankin Scale no apparent weakness of the four extremities, but no spontaneous speech was observed (National Institutes of Health Stroke Scale score = 6). Diffusion-weighted MRI after 9 h after onset disclosed a high intensity lesion from the left anterior limb of the internal capsule to the corona radiata (Table 1, number 4). ECG, echocardiography, and MRI angiography were unremarkable. He was hospitalized that day. On the third day, he was no longer somnolent, but spontaneous speech was still scarce and the voice was weak. He had a moderate degree of aphasia (transcortical motor type: score = 66), but there were no other neurological deficits including hemianopia, and he could walk without assistance. He was calm and somewhat depressive, but obeyed orders by neurologists, nurses, and speech therapists without resistance. In the late afternoon on the fifth hospital day, he went to the radiography department for carotid ultrasonography. After the study, he was instructed to wait for a medical assistant from the neurological ward to pick him up; he was sitting on a wheelchair in front of the radiography department. At that time, his wife who had visited him passed by him saying, "Good bye, I will come and see you tomorrow afternoon."When the medical assistant arrived a few minutes later, the patient was no longer there. One and a half hours later, he arrived uninjured at his own home, about 2 km from the hospital. The next day he went back to the hospital with his wife. When asked about his escape, he mumbled, "I followed my wife. I had forgotten that I was in the hospital." His speech improved significantly 10 days after hospitalization (mild: score = 90). He was discharged from hospital when able to function independently, on the 14th day after onset.

## 2. Discussion

Some patients with acute ischemic stroke who show denial behavior may fail to comprehend that they are unwell, and try to get out and go home [1]. However, escape from hospital to the patient's own home after an acute ischemic stroke has rarely been described previously [4]. Such situations can result in serious injuries to patients and have potential litigation consequences [6]. The exact rate of absconding from hospital in acute stroke patients is not known, but is probably rare. A large study in Hong Kong which analyzed patients with various diseases in a public general hospital reported a **similar low** rate of absconding (0.27%) [3]. It is difficult to draw any definitive conclusions from the present **data** which deals **with** only four patients. However, the present patients had relatively uniform clinical characteristics. They were all male, relatively young (around 60 years old), had moderate-to-severe aphasia without significant motor weakness or hemianopia, and had infarctions in the left MCA territory. It is easy to see how relatively young age and minimum motor deficits can be regarded as requisite conditions for escaping home. Moreover, existence of hemianopia is likely to impede patients' efforts to escape. In moderate-to-severe aphasia, communication with medical staff is very difficult, and lack of recognition of aphasia (anosognosia) can prompt patients to try to get out of bed and go home [1]. In patients with right hemispheric infarction, behavior disorders tend to manifest as delirium or acute confusional state [2,9]; therefore these patients are more prone to falls and may not be able to escape. In addition, hemispatial neglect which is more frequently observed in right hemispheric stroke, is likely to mean that patients trying to escape would not succeed.

In 2002, Hashimoto et al. [4] studied the characteristics of 140 brain injured patients who were determined to be at risks of escape from their ward of the rehabilitation hospital. They found that the group of high-risk patients with such behaviors had better motor function (they could walk or use a wheelchair) and greater impairment in cognitive function, especially orientation, memory, and problem solving than the group of patients without such behaviors. Among the high-risk group, patients who attempted escape but were not successful had more severe cognitive deficits and poorer motor skills than those who could successfully escaped from the ward or hospital. These characteristics correspond well with those of four patients in this study. In the light of these results, Hashimoto et al. [5] attempted several measures to prevent escape such as, improving training for medical staff, installing sensors to detect when patients were away from their bed or room, and attaching wrist bands, name tags, or global positioning systems on patients. Although the percentages of patients who successfully escaped from the ward fell after the introduction of these preventative measures, the percentages of successful escapes from the hospital did not reduce significantly. These patients who successfully escaped from the hospital tended to have better motor skills, and four of seven removed the global positioning system by themselves during their escape.

Although escape from the hospital after acute ischemic stroke is infrequent, patients are at **risk** if they have moderate to severe aphasia, minimum or no motor weakness without hemianopia or hemispatial neglect and relatively young. These patients must therefore be managed cautiously to prevent serious harm.

## References

[1] J. Bogousslavsky, William Feinberg Lecture 2002. Emotions, mood, and behavior after stroke, *Stroke* **34** (2003), 1046–1150.

[2] L. Caeiro, J.M. Ferro, R. Albuquerque and M.L. Figueira, Delirium in the first days of acute stroke, *J Neurol* **251** (2004), 171–178.

[3] S.T. Cheng, C.H. Chung, Y.H. Leung and K.K. Lai, Patient absconding behaviour in a public general hospital: retrospective study, *Hong Kong Med J* **8** (2002), 87–91.

[4] K. Hashimoto, M. Ohashi, S. Watanabe and S. Miyano, Brain injured patients, who have risks of escape from their ward or the hospital: discussion about the characteristics of their impairment and disability, *Jpn J Rehabil Med* **39** (2002), 317–321.

[5] K. Hashimoto, M. Ohashi, M. Kobayashi, T. Kamikubo, S. Watanabe and S. Miyano, Brain injured patients at risk of escape from the ward or hospital: a comparison of patients before and after measures against escape were taken, *Jpn J Rehabil Med* **40** (2003), 369–373.

[6] R.G. Holloway, D. Tuttle, T. Baird and W.K. Skelton, The safety of hospital stroke care, *Neurology* **68** (2007), 550–555.

[7] M.L. Kauhanen, J.T. Korpelainen, P. Hiltunen, R. Määttä, H. Mononen, E. Brusin, K.A. Sotaniemi and V.V. Myllylä, Aphasia, depression, and non-verbal cognitive impairment in ischaemic stroke, *Cerebrovasc Dis* **10** (2000), 455–461.

[8] N. Kerse, V. Parag, V.L. Feigin, H. McNaughton, M.L. Hackett, D.A. Bennett, C.S. Anderson and the Auckland Regional Community Stroke (ARCOS) Study Group, Falls after stroke. Results from the Auckland Regional Community Stroke (ARCOS) Study, 2002 to 2003, *Stroke* **39** (2008), 1890–1893.

[9] E. Mori and A. Yamadori, Acute confusional state and acute agitated delirium. Occurrence after infarction in the right middle cerebral artery territory, *Arch Neurol* **44** (1987), 1139–1143.
